# Supplementary material for: Insecticide-treated net (ITN) use, factors associated with non-use of ITNs, and occurrence of sand flies in three communities with reported cases of cutaneous leishmaniasis in Ghana
Source: PLoS One. 2021 Dec 16;16(12):e0261192. doi: 10.1371/journal.pone.0261192 (PMC8675665; doi:10.1371/journal.pone.0261192)
Supplement: S1 File — (DOCX) [file pone.0261192.s001.docx]

### **Household characteristics**

A total of 536 (91.3%) out of the 587 households had electricity within their households with 178 (94.2%), 185 (92.5%), and 173 (87.4%) households having electricity in Ashiabre, Keri and Sibi Hilltop respectively (Table 1).

The main material of the household dwelling floor observed in 425 (72.4%) of the households was cement, followed by earth/sand 96 (16.4%) and carpet 55 (9.4%) (12). In the study communities, 81.0%, 69.5%, and 67.2% of household dwellings in Ashiabre, Keri, and Sibi Hilltop recorded cement as the main material of the dwelling floor (Table 1).

Majority (90.8%) of the household dwelling across the study communities had metallic roofing while the main material of the exterior wall of the household dwellings of all study communities were cement blocks for 136 (23.2%) households, mud with cement covering for 162 (27.6%) households, stone with mud covering for 149 (25.4%) households, and bamboo with mud covering for 140 (23.9%) households.

In Ashiabre, 34.9% of the household dwellings had exterior wall made of stone with mud covering while 36.0% of household dwellings in Keri had exterior walls made of bamboo with mud covering. In Sibi Hilltop, 27.6% of the household dwellings had exterior wall made of mud with cement covering (Table 1).

Most of the households (71%) in the study communities used 2-5 rooms for sleeping which ranged from 1 to 18 in Ashiabre and Keri but was 1 to 12 in Sibi Hilltop (Table 1). The number of household windows ranged from 0 to 15 with Ashiabre having a range of 0 to 13 household windows, while Keri had a range of 1 to 15 household widows, and Sibi Hilltop recorded a range of 0 to 12 household windows. Overall, majority (59.1%) of household dwellings had 2 to 4 windows. This observation was consistent across the study communities as 53.4%, 57.0%, and 66.7% of household dwellings in Ashiabre, Keri, and Sibi Hilltop respectively also recorded 2 to 4 windows (Table 2). Most households (71%) had windows with no screen/nets.

Most of the households (58%) cooked outdoors and the remaining 42% used a separate room as kitchen distributed as follows: Keri and Sibi Hilltop 65.0% and 62.1% cooked outdoors and 55.0% used a separate room as kitchen at Ashiabre.

Wood was the main source of fuel used for cooking in most (91.5%) of the study households with Ashiabre, Keri, and Sibi Hilltop recording 97.9%, 85.0%, and 91.9% respectively (Table 2).

Regarding main source of drinking water and toilet facility, 51% of the households used surface water and 84% lacked toilet facility and so used the bush/field (Table 3).

The use of surface water as main source of drinking water was particularly high among households in Ashiabre (61.9%) and Sibi Hilltop (79.3%). In Keri however, majority (64.5%) of the study households used public tap/standpipe as their main source of drinking water (Table 3).

Table 1: Characteristics of study households

|  | | | | | |
| --- | --- | --- | --- | --- | --- |
| Housing Characteristics | Categories | Study Communities | | | |
|  |  | Ashiabre | Keri | Sibi Hill Top | Total |
|  |  | n (%) | n (%) | n (%) | n (%) |
|  |  |  |  |  |  |
| Electricity | Have Electricity | 178 (94.2) | 185 (92.5) | 173 (87.4) | 536 (91.3) |
|  | No Electricity | 11 (5.8) | 15 (7.5) | 25 (12.6) | 51 (8.7) |
|  | Subtotal | 189 (100) | 200 (100) | 198 (100) | 587 (100) |
| Main material of the dwelling floor |  |  |  |  |  |
|  | Earth / Sand | 12 (6.3) | 44 (22.0) | 40 (20.2) | 96 (16.4) |
|  | Dung | 1 (0.5) | 2 (1.0) | 3 (1.5) | 6 (1.0) |
|  | Wood planks | 0 (0) | 1 (0.5) | 1 (0.5) | 2 (0.3) |
|  | Ceramic tiles | 1 (0.5) | 0 (0) | 2 (1.0) | 3 (0.5) |
|  | Cement | 153 (81.0) | 139 (69.5) | 133 (67.2) | 425 (72.4) |
|  | Carpet | 22 (11.6) | 14 (7) | 19 (9.6) | 55 (9.4) |
|  | Subtotal | 189 (100) | 200 (100) | 198 (100) | 587 (100) |
| Main material of the roof |  |  |  |  |  |
|  | Thatch / Palm leaf | 11 (5.8) | 18 (9.0) | 25 (12.6) | 54 (9.2) |
|  | Metal | 178 (94.2) | 182 (91.0) | 173 (87.4) | 533 (90.8) |
|  | Subtotal | 189 (100) | 200 (100) | 198 (100) | 587 (100) |
| Main material of exterior wall |  |  |  |  |  |
|  | Bamboo with mud | 26 (13.8) | 72 (36.0) | 42 (21.2) | 140 (23.9) |
|  | Stone with mud | 52 (27.5) | 33 (16.5) | 64 (32.3) | 149 (25.4) |
|  | Mud with cement | 66 (34.9) | 42 (21.0) | 54 (27.3) | 162 (27.6) |
|  | Cement blocks | 45 (23.8) | 53 (26.5) | 38 (19.2) | 136 (23.2) |
|  | Subtotal | 189 (100) | 200 (100) | 198 (100) | 587 (100) |
| Rooms used for sleeping |  |  |  |  |  |
|  | 1 | 20 (10.6) | 25 (12.5) | 16 (8.10 | 61 (10.4) |
|  | 2 to 5 | 126 (66.7) | 137 (68.5) | 151 (76.3) | 414 (70.5) |
|  | 6 to 10 | 36 (19.1) | 33 (16.5) | 27 (13.6) | 96 (16.4) |
|  | > 10 | 7 (3.7) | 5 (2.5) | 4 (2.0) | 16 (2.7) |
|  | Subtotal | 189 (100) | 200 (100) | 198 (100) | 587 (100) |

**Table 2:** Characteristics of study households (continuation)

| Housing Characteristics | Categories | Study Communities | | | |
| --- | --- | --- | --- | --- | --- |
|  |  | Ashiabre | Keri | Sibi Hill Top | Total |
|  |  | n (%) | n (%) | n (%) | n (%) |
|  |  |  |  |  |  |
| Household number of windows |  |  |  |  |  |
|  | No windows | 3 (1.6) | 0 (0) | 1 (0.5) | 4 (0.7) |
|  | 1 window | 17 (9.0) | 27 (13.5) | 15 (7.6) | 59 (10.1) |
|  | 2 to 4 windows | 101 (53.4) | 114 (57.0) | 132 (66.7) | 347 (59.1) |
|  | 5 to 7 windows | 46 (24.3) | 44 (22.0) | 36 (18.2) | 126 (21.5) |
|  | 8 to 10 windows | 16 (8.5) | 11 (5.5) | 11 (5.6) | 38 (6.5) |
|  | > 10 windows | 6 (3.2) | 4 (2.0) | 3 (1.5) | 13 (2.2) |
|  | Subtotal | 189 (100) | 200 (100) | 198 (100) | 587 (100) |
| Windows with screen/net |  |  |  |  |  |
|  | No screened windows | 118 (62.4) | 140 (70.0) | 158 (79.8) | 416 (70.9) |
|  | 1 screened window | 17 (9.0) | 13 (6.5) | 9 (4.5) | 39 (6.6) |
|  | 2 to 4 screened windows | 37 (19.6) | 32 (16.0) | 23 (11.6) | 92 (15.7) |
|  | 5 to 7 screened windows | 10 (5.3) | 11 (5.5) | 5 (2.5) | 26 (4.4) |
|  | 8 to 10 screened windows | 6 (3.2) | 4 (2) | 3 (1.5) | 13 (2.2) |
|  | > 10 screened windows | 1 (0.5) | 0 (0) | 0 (0) | 1 (0.2) |
|  | Subtotal | 189 (100) | 200 (100) | 198 (100) | 587 (100) |
| Place for cooking |  |  |  |  |  |
|  | In a separate room used an kitchen | 104 (55.0) | 70 (35.0) | 75 (37.9) | 249 (42.4) |
|  | Outdoors | 85 (45.0) | 130 (65.0) | 123 (62.1) | 338 (57.6) |
|  | Subtotal | 189 (100) | 200 (100) | 198 (100) | 587 (100) |
| Main cooking fuel |  |  |  |  |  |
|  | Kerosene | 0 (0) | 18 (9.0) | 5 (2.5) | 23 (3.9) |
|  | Charcoal | 4 (2.1) | 12 (6.0) | 11 (5.6) | 27 (4.6) |
|  | Wood | 185 (97.9) | 170 (85.0) | 182 (91.9) | 537 (91.5) |
|  | Subtotal | 189 (100) | 200 (100) | 198 (100) | 587 (100) |

**Table 3:** Household main source of drinking water and toilet facility

|  |  | **Study Communities** | |  |  |
| --- | --- | --- | --- | --- | --- |
| **Characteristics** | **Categories** | **Ashiabre** | **Keri** | **Sibi Hill Top** | **Total** |
|  |  | **n (%)** | **n (%)** | **n (%)** | **n (%)** |
| **Main Source of Drinking water** |  |  |  |  |  |
|  | **Improved source** |  |  |  |  |
|  | Public tap / standpipe | 50 (26.5) | 129 (64.5) | 18 (9.1) | 197 (33.6) |
|  | Tube Well, Borehole | 14(7.4) | 43 (21.5) | 2 (1) | 59 (10.1) |
|  | Protected well | 2 (1.1) | 1 (0.5) | 16 (8.1) | 19 (3.2) |
|  | **Non-improved source** |  |  |  |  |
|  | Unprotected well | 6 (3.2) | 0 (0) | 5 (2.5) | 11 (1.9) |
|  | Surface water | 117 (61.9) | 27 (13.5) | 157 (79.3) | 301 (51.3) |
|  | Subtotal | 189 (100) | 200 (100) | 198 (100) | 587 (100) |
| **Main type of toilet facility** |  |  |  |  |  |
|  | **Improved facility** |  |  |  |  |
|  | Ventilated Improved Pit latrine (VIP) | 13 (6.9) | 5 (2.5) | 5 (2.5) | 23 (3.9) |
|  | Pit latrine with slab | 24 (12.7) | 19 (9.5) | 4 (2) | 47 (8) |
|  | **Non-improved facility** |  |  |  |  |
|  | Pit latrine without slab / Open pit | 12 (6.3) | 9 (4.5) | 4 (2) | 25 (4.3) |
|  | No facility, bush, field | 140 (74.1) | 167 (83.5) | 185 (93.4) | 492 (83.8) |
|  | Subtotal | 189 (100) | 200 (100) | 198 (100) | 587 (100) |

#### **Household possessions**

Table 4 below shows that radios (47.2%), televisions (50.3%) and mobile telephones (80.4%) were common durable goods owned by the study households. Mobile telephones were particularly more common among the households and were owned by at least one person in 164 (86.8%), 134 (67.0%), and 174 (87.9%) households in Ashiabre, Keri and Sibi Hilltop, respectively. Overall, ownership of refrigerator was observed in only 6.5% of the study households (Table 4).

Regarding ownership of means of transportation, bicycle ownership was the majority (74.8%). In the respective communities, 88.4%, 63.0%, and 73.7% of households in Ashiabre, Keri, and Sibi Hilltop owned at least one bicycle as a means of transportation (Table 4).

Ownership of agricultural land was observed in 72.7% of the households. In the study communities, agricultural land was owned by 69.3%, 58.5%, and 90.4% of households in Ashiabre, Keri, and Sibi Hilltop respectively (Table 4).

Farm animals were owned by 174 (92.1%) households in Ashiabre. In Keri and Sibi Hilltop, 126 (63.0%), and 167 (84.3%) households owned farm animals. Cumulatively, 79.6% of the study households owned at least one farm animal (Table 4).

**Table 4**: Summary of household possessions

|  | Study Communities | | | |
| --- | --- | --- | --- | --- |
|  | Ashiabre | Keri | Sibi Hill Top | Total |
| Possession | n (%) | n (%) | n (%) | n (%) |
|  |  |  |  |  |
| Household effects |  |  |  |  |
| Radio | 100 (52.9) | 88 (44.0) | 89 (44.9) | 277 (47.2) |
| Television | 108 (57.1) | 92 (46.0) | 95 (48) | 295 (50.3) |
| Mobile telephone | 164 (86.8) | 134 (67.0) | 174 (87.9) | 472 (80.4) |
| Non-mobile telephone | 35 (18.5) | 23 (11.5) | 8 (4) | 66 (11.2) |
| Refrigerator | 17 (9) | 6 (3.0) | 15 (7.6) | 38 (6.5) |
| Means of transport |  |  |  |  |
| Bicycle | 167 (88.4) | 126 (63.0) | 146 (73.7) | 439 (74.8) |
| Animal drawn cart | 23 (12.2) | 33 (16.5) | 33 (16.7) | 89 (15.2) |
| Motorcycle/scooter | 95 (50.3) | 57 (28.5) | 76 (38.4) | 228 (38.8) |
| Car/truck | 4 (2.1) | 2 (1.0) | 7 (3.5) | 13 (2.2) |
| Boat with motor | 3 (1.6) | 1 (0.5) | 5 (2.5) | 9 (1.5) |
| Boat without motor | 3 (1.6) | 2 (1.0) | 0 (0) | 5 (0.9) |
|  |  |  |  |  |
| Ownership of agricultural land | 131 (69.3) | 117 (58.5) | 179 (90.4) | 427 (72.7) |
| Ownership of farm animals ^1^ | 174 (92.1) | 126 (63) | 167 (84.3) | 467 (79.6) |
|  |  |  |  |  |
| Number of households | 189 (100) | 200 (100) | 198 (100) | 587 (100) |
|  |  |  |  |  |
| ^1^ Cattle, milk cows, goats, sheep, chicken, ducks, other poultry | | | | |
