# Supplementary material for: Insecticide-treated net (ITN) use, factors associated with non-use of ITNs, and occurrence of sand flies in three communities with reported cases of cutaneous leishmaniasis in Ghana
Source: PLoS One. 2021 Dec 16;16(12):e0261192. doi: 10.1371/journal.pone.0261192 (PMC8675665; doi:10.1371/journal.pone.0261192)
Supplement: S2 File — (DOCX) [file pone.0261192.s002.docx]

STROBE Statement— **Insecticide-treated net (ITN) use, factors associated with non-use of ITNs, and occurrence of sand flies in three communities with reported cases of cutaneous leishmaniasis in Ghana.**

|  | **Item No** | **Recommendation** | **Page No.** | **Relevant text from manuscript** |
| --- | --- | --- | --- | --- |
| **Title and abstract** | 1 | (*a*) Indicate the study’s design with a commonly used term in the title or the abstract | 2 | Line 29-30; Using a cross-sectional study design |
|  |  | (*b*) Provide in the abstract an informative and balanced summary of what was done and what was  found | 2 | Lines (27-46); Abstract |
| **Introduction** | | |  |  |
| Background/rationale | 2 | Explain the scientific background and rationale for the investigation being reported | 4 | Introduction (Lines 51-103) |
| Objectives | 3 | State specific objectives, including any prespecified hypotheses | 5 | Introduction Lines (101-103); This study was therefore conducted as part of a larger study investigating *Leishmania* infection and ITN use in three communities of the Oti region of Ghana, to obtain data on ITN use, factors associated with non-use of ITNs, and the occurrence of sand flies. |
| **Methods** | | |  |  |
| Study design | 4 | Present key elements of study design early in the paper | 5 | Methods, Lines (111-117); Using a cross-sectional study design, this study was conducted in three communities of the Oti region of Ghana from October to December 2018. |
| Setting | 5 | Describe the setting, locations, and relevant dates, including periods of recruitment, exposure, follow-up, and data collection | 5 | Methods, Lines (118 - 131); This study was conducted in the following three communities: Ashiabre, Keri, and Sibi Hilltop. Ashiabre is in the Tutukpene sub-district of the Nkwanta South municipality while Keri is in the Keri sub-district of the municipality |
| Participants | 6 | (*a*) *Cohort study*—Give the eligibility criteria, and the sources and methods of selection of participants. Describe methods of follow-up  *Case-control study*—Give the eligibility criteria, and the sources and methods of case |  |  |

|  |  | ascertainment and control selection. Give the rationale for the choice of cases and controls  *Cross-sectional study*—Give the eligibility criteria, and the sources and methods of selection of participants | 6 | Methods, Line 133 to 151; Eligible study participants were household heads who were residents in the study community for > 12 months. |
| --- | --- | --- | --- | --- |
|  |  | (*b*) *Cohort study*—For matched studies, give matching criteria and number of exposed and unexposed  *Case-control study*—For matched studies, give matching criteria and the number of controls per  case | N/A | This was a cross-sectional study |
| Variables | 7 | Clearly define all outcomes, exposures, predictors, potential confounders, and effect modifiers. Give diagnostic criteria, if applicable | 7-8 | Methods, Lines 160 - 195 |
| Data sources/ measurement | 8* | For each variable of interest, give sources of data and details of methods of assessment (measurement). Describe comparability of assessment methods if there is more than one group | 5 | Methods, Line 114 to 117; ITN ownership, access, use, and factors associated with non-use of ITN were investigated through a household survey. The occurrence of sand flies in the following locations of each study community was also investigated using CDC light traps (outdoor) and indoor aspiration: households, school, church, and mosque. |
| Bias | 9 | Describe any efforts to address potential sources of bias | 6 | Lines (151); Details of household selection procedure for this study is published |

|  |  |  |  |  |
| --- | --- | --- | --- | --- |
| Study size | 10 | Explain how the study size was arrived at | 6. | Methods. Line 143 to 151;  Sample size consideration. |
| Quantitative variables | 11 | Explain how quantitative variables were handled in the analyses. If applicable, describe which groupings were chosen and why | 9 | Methods, Line 197 - 247;  Data Management and analysis |
| Statistical methods | 12 | (*a*) Describe all statistical methods, including those used to control for confounding | 9 | Methods, Line 238 to 247; |
|  |  | (*b*) Describe any methods used to examine subgroups and interactions | N/A | There were no subgroups |
|  |  | (*c*) Explain how missing data were addressed | N/A | There was no missing data |
|  |  | (*d*) *Cohort study*—If applicable, explain how loss to follow-up was addressed  *Case-control study*—If applicable, explain how matching of cases and controls was addressed *Cross-sectional study*—If applicable, describe analytical methods taking account of sampling strategy | 7 | Methods: Lines 152-154); |
|  |  | (*e*) Describe any sensitivity analyses | N/A |  |
| Results |  |  |  |  |
| Participants | 13* | (a) Report numbers of individuals at each stage of study—eg numbers potentially eligible,  examined for eligibility, confirmed eligible, included in the study, completing follow-up, and analysed | N/A | This was a cross-sectional study with only one stage |
|  |  | (b) Give reasons for non-participation at each stage | N/A |  |
|  |  | (c) Consider use of a flow diagram | N/A |  |
| Descriptive data | 14* | (a) Give characteristics of study participants (eg demographic, clinical, social) and information on exposures and potential confounders | 10 | Results, Line 254 - 270; |

|  |  |  |  |  |
| --- | --- | --- | --- | --- |
|  |  | (b) Indicate number of participants with missing data for each variable of interest | N/A | There was no missing data |
|  |  | (c) *Cohort study*—Summarise follow-up time (eg, average and total amount) | N/A | This was a cross-sectional study |
| Outcome data | 15* | *Cohort study*—Report numbers of outcome events or summary measures over time | N/A | This was a cross-sectional study |
|  |  | *Case-control study—*Report numbers in each exposure category, or summary measures of exposure | N/A | This was a cross-sectional study |
|  |  | *Cross-sectional study—*Report numbers of outcome events or summary measures | 10-18. | Results, Line 297 to 445; |
| Main results | 16 | (*a*) Give unadjusted estimates and, if applicable, confounder-adjusted estimates and their precision (eg, 95% confidence interval). Make clear which confounders were adjusted for and why they were included | 12. | Results, Line 442 to 445. |
|  |  | (*b*) Report category boundaries when continuous variables were categorized | N/A | There were no continuous variables. |
|  |  | (*c*) If relevant, consider translating estimates of relative risk into absolute risk for a meaningful  time period | N/A | There were no estimates of relative risk |
| Other analyses | 17 | Report other analyses done—eg analyses of subgroups and interactions, and sensitivity analyses | N/A | There were no subgroups |
| **Discussion** |  |  |  |  |
| Key results | 18 | Summarise key results with reference to study objectives | 22. | Conclusion, Line 607 to 613 |
| Limitations | 19 | Discuss limitations of the study, taking into account sources of potential bias or imprecision.  Discuss both direction and magnitude of any potential bias | 22 | Limitation, Line 615 to 620 |
| Interpretation | 20 | Give a cautious overall interpretation of results considering objectives, limitations, multiplicity of analyses, results from similar studies, and other relevant evidence | 22 | Conclusion, Line 607 to 613 |

| Generalisability | 21 | Discuss the generalisability (external validity) of the study results | 21 | Discussion, Line 479-605; |
| --- | --- | --- | --- | --- |
| **Other information** |  |  |  |  |
| Funding | 22 | Give the source of funding and the role of the funders for the present study and, if applicable, for the original study on which the present article is based | 23 | Lines 623-625; The authors are grateful to the TDR post graduate scheme in implementation science studentship support at the School of Public Health, University Ghana for funding to conduct this study. |
