# Supplementary material for: Insecticide-treated net (ITN) use, factors associated with non-use of ITNs, and occurrence of sand flies in three communities with reported cases of cutaneous leishmaniasis in Ghana
Source: PLoS One. 2021 Dec 16;16(12):e0261192. doi: 10.1371/journal.pone.0261192 (PMC8675665; doi:10.1371/journal.pone.0261192)
Supplement: S3 File — (PDF) [file pone.0261192.s003.pdf]

HOUSEHOLD QUESTIONNAIRE

HOUSEHOLD INFORMATION PANEL	HH
HH1A. Cluster number: ___ ___ ___<br>HH1B. Community name: ______________________	HH2. Household number: __ __ __ | __ __ __
HH3. Interviewer name and number:<br>Name _________________________ ___ ___	HH4. Supervisor name and number:<br>Name__________________________ ___ ___
HH5. Day / Month / Year of interview: ___ ___ / ___ ___ / ___ ___ ___ ___	
HH6. Area:<br>Urban ....1<br>Rural...2	HH7. District: Ho 1

Allow respondent to the Household Questionnaire enough time to read the household consent form or to have it read to him/her before proceeding further with the questionnaire administration.

Is permission given?

- ☐ *Yes, permission is given* ⇨ *Go to HH15 (on next page) to record the time and then begin the interview.*
- ☐ *No, permission is not given* ⇨ *Complete HH9. Discuss this result with your supervisor.*

After all questionnaires for the household have been completed, fill in the following information:	
HH8. Name of head of household:	____
HH9. Result of household interview:<br>Completed ....01<br>No household member or no competent respondent at home at time of visit .... 02<br>Entire household absent for extended period of time... 03<br>Refused .... 04<br>Dwelling vacant / Address not a dwelling....... 05<br>Dwelling destroyed .... 06<br>Dwelling not found .... 07<br>Other (*specify*) ________________________ 96	HH10. Respondent to household questionnaire:<br>Name:_______________________________________<br>Line Number: ___ ___<br><br>HH11. Total number of household members: ___ ___
HH12. Number of women age 15-49 years: ___ ___	HH13. Number of under-5s : ___ ___

HH15.
Record the time.

Hours ....__ __

Minutes ......__ __

HOUSEHOLD LISTING FORM HL

FIRST, PLEASE TELL ME THE NAME OF EACH PERSON WHO USUALLY LIVES HERE, STARTING WITH THE HEAD OF THE HOUSEHOLD.

List the head of the household in line 01. List names of all household members (HL2), their relationship to the household head (HL3), and their sex (HL4)

Then ask: ARE THERE ANY OTHERS WHO LIVE HERE, EVEN IF THEY ARE NOT AT HOME NOW?

If yes, complete listing for questions HL2-HL4. Then, ask questions starting with HL5 for each person at a time.

	For household members age **15 and above**	Women age **15-49**	For children **under 5 years** of age	For all household members

HL1. *Line no.*	HL2. *Name*	HL3. WHAT IS THE RELATION-SHIP OF (*name*) TO THE HEAD OF HOUSE-HOLD?	HL4. IS (*name*) MALE OR FEMALE ?<br>1 Male<br>2 Female	HL5. WHAT IS (*name*)'s DATE OF BIRTH?<br>98 DK 9998 DK<br>*Ensure that every person less than 6 years of age has a month and year of birth*		HL6. HOW OLD IS (*name*)?<br>*Record in completed years. If age is 95 or above, record '95'*	HL7. WHAT IS (*name*)'s CURRENT MARITAL STATUS?<br>1 Married or living together<br>2 Divorced/ separated<br>3 Widowed<br>4 Never married or lived together	HL8.<br>*Circle line number if woman is age* **15-49**	HL9<br>*Circle line number if child is* **under 5 years** *of age*	HL10. WHAT IS (*name*)'s NATIONALITY?<br>*Insert appropriate nationality code*	HL11. WHERE WAS (*name*) BORN?<br>IN THIS VILLAGE OR TOWN?	ELSEWHERE?<br>*Insert village or town name and appropriate birthplace code*	HL12. FOR HOW MANY YEARS HAS (*name*) LIVED IN THIS VILLAGE/ TOWN?<br>*Record zero if less than one year*
Line	Name	Relation	M F	MM	Year	Age	Marital status	15-49	Under 5	Nationality	Y N	Village/Town Code	Years
01		0 1	1 2	_ _	_ _ _ _	__ __	1 2 3 4	01	01	— —	1 2→	________|__ __	__ __
02		__ __	1 2	_ _	_ _ _ _	__ __	1 2 3 4	02	02	— —	1 2→	________|__ __	__ __
03		__ __	1 2	_ _	_ _ _ _	__ __	1 2 3 4	03	03	— —	1 2→	________|__ __	__ __
04		__ __	1 2	_ _	_ _ _ _	__ __	1 2 3 4	04	04	— —	1 2→	________|__ __	__ __
05		__ __	1 2	_ _	_ _ _ _	__ __	1 2 3 4	05	05	— —	1 2→	________|__ __	__ __
06		__ __	1 2	_ _	_ _ _ _	__ __	1 2 3 4	06	06	— —	1 2→	________|__ __	__ __
07		__ __	1 2	_ _	_ _ _ _	__ __	1 2 3 4	07	07	— —	1 2→	________|__ __	__ __
08		__ __	1 2	_ _	_ _ _ _	__ __	1 2 3 4	08	08	— —	1 2→	________|__ __	__ __

HL1. *Line no.*	HL2. *Name*	HL3. WHAT IS THE RELATION-SHIP OF (*name*) TO THE HEAD OF HOUSE-HOLD?	HL4. IS (*name*) MALE OR FEMALE ? 1 Male 2 Female	HL5. WHAT IS (*name*)'s DATE OF BIRTH? 98 DK 9998 DK *Ensure that every person less than 6 years of age has a month and year of birth*		HL6. HOW OLD IS (*name*)? *Record in completed years. If age is 95 or above, record '95'*	HL7. WHAT IS (*name*)'s CURRENT MARITAL STATUS? 1 Married or living together 2 Divorced/ separated 3 Widowed 4 Never married or lived together	HL8. *Circle line number if woman is age* ***15-49***	HL9 *Circle line number if child is* ***under 5 years*** *of age*	HL10. WHAT IS (*name*)'s NATIONALITY? *Insert appropriate nationality code*			HL11. WHERE WAS (*name*) BORN? IN THIS VILLAGE OR TOWN?	HL11. ELSEWHERE? *Insert village or town name and appropriate birthplace code*	HL12. FOR HOW MANY YEARS HAS (*name*) LIVED IN THIS VILLAGE/ TOWN? *Record zero if less than one year*
Line	Name	Relation	M F	MM	Year	Age	Marital status	15-49	Under 5	Nationality			Y N	Village/Town Code	Years
09		__ __	1 2	__ __	__ __ __ __	__ __	1 2 3 4	09	09	— —			1 2→	________ | __ __	__ __
10		__ __	1 2	__ __	__ __ __ __	__ __	1 2 3 4	10	10	— —			1 2→	________ | __ __	__ __
11		__ __	1 2	__ __	__ __ __ __	__ __	1 2 3 4	11	11	__ __	1 2 3 8	— —	1 2→	________ | __ __	__ __
12		__ __	1 2	__ __	__ __ __ __	__ __	1 2 3 4	12	12	__ __	1 2 3 8	— —	1 2→	________ | __ __	__ __
13		__ __	1 2	__ __	__ __ __ __	__ __	1 2 3 4	13	13	__ __	1 2 3 8	— —	1 2→	________ | __ __	__ __
14		__ __	1 2	__ __	__ __ __ __	__ __	1 2 3 4	14	14	__ __	1 2 3 8	— —	1 2→	________ | __ __	__ __
15		__ __	1 2	__ __	__ __ __ __	__ __	1 2 3 4	15	15	__ __	1 2 3 8	— —	1 2→	________ | __ __	__ __

Tick here if additional questionnaire used ☐

Probe for additional household members.
Probe especially for any infants or small children not listed, and others who may not be members of the family (such as servants, friends) but who usually live in the household.

** Codes for HL3: Relationship to head of household: ....*

01 Head	09 Brother-In-Law / Sister-In-Law
02 Wife / Husband	10 Uncle / Aunt
03 Son / Daughter	11 Niece / Nephew
04 Son-In-Law / Daughter-In-Law	12 Other relative
05 Grandchild	13 Adopted / Foster / Stepchild
06 Parent	14 Not related
07 Parent-In-Law	98 Don't know
08 Brother / Sister	

Codes for HL10: Nationality

01 Ghanaian by birth
02 Ghanaian by naturalisation
03 Dual nationality (Ghanaian & other)
04 African
05 European
06 American (North, South & Caribbean
07 Asian & Oceanian (incl. Australia & New Zealand)
98 Don’t know

Codes for HL11: Birthplace

01 Western region	09 Upper East
02 Central region	10 Upper West
03 Greater Accra	11 Africa
04 Volta region	12 Europe
05 Eastern region	13 Americas (North, South & Caribbean)
06 Ashanti region	14 Asia
07 Brong Ahafo region	15 Oceania
08 Northern region	98 Don’t know

RESIDENCE AND EDUCATION ED

		For all household members			For those **11years of age and above**	For those **5 years of age and above**					
ED1.<br>*Line no.*	ED2.<br>*Name and age*<br>*Copy from Household Listing Form, HL2 and HL6*	ED3.<br>DID (*name*) STAY HERE LAST NIGHT?<br>1 Yes<br>2 No	ED4.<br>DID (*name*) LIVE HERE MOST OF THE LAST 6 MONTHS?<br>1 Yes<br>2 No	ED5.<br>DOES (*name*) EXPECT TO LIVE HERE FOR MOST OF THE NEXT 6 MONTHS?<br>**1 Yes**<br>**2 No**<br>**8 DK**	ED6.<br>IN WHAT LANGUAGE CAN (*name*) READ AND WRITE?<br>Code:<br>1 None (not literate)<br>2 English only<br>3 Ghanian only<br>4 English & Ghanaian<br>5 English & French<br>6 Other language than Ghanaian, English or French<br>8 DK	ED7.<br>HAS (*name*) EVER ATTENDED SCHOOL OR PRE-SCHOOL?<br>1 Yes<br>2 No ⇘ *Next line*	ED8.<br>DURING THE PRESENT SCHOOL YEAR, DID (*name*) ATTEND SCHOOL OR PRESCHOOL AT ANY TIME?<br>1 Yes ⇨ED10<br>2 No ⇨ED9	ED9.<br>WHAT IS THE HIGHEST LEVEL OF SCHOOL (*name*) ATTENDED?<br>WHAT IS HIGHEST GRADE (*name*) COMPLETED AT THIS LEVEL?<br>Level:<br>0 Preschool<br>1 Primary<br>2 Junior HS<br>3 Senior HS<br>4 Higher<br>8 DK<br>*If level=0, skip to next line*	Grade:<br>98 DK<br>*If less than 1 grade, enter 00.*	ED10.<br>DURING THIS SCHOOL YEAR, WHICH LEVEL AND GRADE IS (*name*) ATTENDING?<br>Level:<br>0 Preschool<br>1 Primary<br>2 Junior HS<br>3 Senior HS<br>4 Higher<br>8 DK<br>*If level=0, skip to next line*	Grade<br>98 DK

Line	Name	Age	Yes No	Yes No	Yes No DK	Literacy code	Yes No	Yes No	Level	Grade	Level	Grade
01		__ __	1 2	1 2	1 2 8	1 2 3 4 5 6 8	1 2	1⇨ED10 2	0 1 2 3 4 8	__ __ *Next line*	0 1 2 3 4 8	__ __ *Next line*
02		__ __	1 2	1 2	1 2 8	1 2 3 4 5 6 8	1 2	1⇨ED10 2	0 1 2 3 4 8	__ __ *Next line*	0 1 2 3 4 8	__ __ *Next line*
03		__ __	1 2	1 2	1 2 8	1 2 3 4 5 6 8	1 2	1⇨ED10 2	0 1 2 3 4 8	__ __ *Next line*	0 1 2 3 4 8	__ __ *Next line*
04		__ __	1 2	1 2	1 2 8	1 2 3 4 5 6 8	1 2	1⇨ED10 2	0 1 2 3 4 8	__ __ *Next line*	0 1 2 3 4 8	__ __ *Next line*
05		__ __	1 2	1 2	1 2 8	1 2 3 4 5 6 8	1 2	1⇨ED10 2	0 1 2 3 4 8	__ __ *Next line*	0 1 2 3 4 8	__ __ *Next line*
06		__ __	1 2	1 2	1 2 8	1 2 3 4 5 6 8	1 2	1⇨ED10 2	0 1 2 3 4 8	__ __ *Next line*	0 1 2 3 4 8	__ __ *Next line*
07		__ __	1 2	1 2	1 2 8	1 2 3 4 5 6 8	1 2	1⇨ED10 2	0 1 2 3 4 8	__ __ *Next line*	0 1 2 3 4 8	__ __ *Next line*
08		__ __	1 2	1 2	1 2 8	1 2 3 4 5 6 8	1 2	1⇨ED10 2	0 1 2 3 4 8	__ __ *Next line*	0 1 2 3 4 8	__ __ *Next line*
09		__ __	1 2	1 2	1 2 8	1 2 3 4 5 6 8	1 2	1⇨ED10 2	0 1 2 3 4 8	__ __ *Next line*	0 1 2 3 4 8	__ __ *Next line*
10		__ __	1 2	1 2	1 2 8	1 2 3 4 5 6 8	1 2	1⇨ED10 2	0 1 2 3 4 8	__ __ *Next line*	0 1 2 3 4 8	__ __ *Next line*
11		__ __	1 2	1 2	1 2 8	1 2 3 4 5 6 8	1 2	1⇨ED10 2	0 1 2 3 4 8	__ __ *Next line*	0 1 2 3 4 8	__ __ *Next line*
12		__ __	1 2	1 2	1 2 8	1 2 3 4 5 6 8	1 2	1⇨ED10 2	0 1 2 3 4 8	__ __ *Next line*	0 1 2 3 4 8	__ __ *Next line*

NO.	QUESTIONS AND FILTERS	CODING CATEGORIES	SKIP
001	Does your household have any mosquito nets that can be used while sleeping?	YES . . . . 1....<br>NO . . . . 2....	 136A
002	How many mosquito nets does your household have?<br><br>IF 7 OR MORE NETS, RECORD '7'.	NUMBER OF NETS . . . . [ ]	

		NET #1	NET #2	NET #3
003	ASK THE RESPONDENT TO SHOW YOU ALL THE NETS IN THE HOUSEHOLD<br><br>IF MORE THAN 3 NETS, USE ADDITIONAL QUESTIONNAIRE(S).	OBSERVED HANGING . . . . 1<br>OBSERVED NOT HANGING OR PACKAGED . . . 2<br>NOT OBSERVED . . 3	OBSERVED HANGING . . . . 1<br>OBSERVED NOT HANGING OR PACKAGED . . . 2<br>NOT OBSERVED . . 3	OBSERVED HANGING . . . . 1<br>OBSERVED NOT HANGING OR PACKAGED . . . . . 2<br>NOT OBSERVED . . . 3
004	How many months ago did your household get the mosquito net?<br>IF LESS THAN ONE MONTH AGO, RECORD '00'.	MONTHS AGO . . . [ ][ ]<br>MORE THAN 36 MONTHS AGO . 95<br>NOT SURE 98	MONTHS AGO . . . [ ][ ]<br>MORE THAN 36 MONTHS AGO . 95<br>NOT SURE 98	MONTHS AGO . . . [ ][ ]<br>MORE THAN 36 MONTHS AGO . . 95<br>NOT SURE 98
005	Where did you get this net?	**PUBLIC SECTOR**<br>GOVT. HOSPITAL/ POLYCLINIC . . . 11<br>GOVT. HEALTH CENTE . . . . 12<br>GOVT. HEALTH POST/CHPS . . . 13<br>FIELDWORKER/	**PUBLIC SECTOR**<br>GOVT. HOSPITAL/ POLYCLINIC . . . 11<br>GOVT. HEALTH CENTER . . . . 12<br>GOVT. HEALTH POST/CHPS . . . 13<br>FIELDWORKER/	**PUBLIC SECTOR**<br>GOVT. HOSPITAL/ POLYCLINIC . . . . . 11<br>GOVT. HEALTH CENTE . . . . 12<br>GOVT. HEALTH POST/CHPS . . . . . 13<br>FIELDWORKER/

			OUTREACH/ PEER<br>EDUCATOR 14	OUTREACH/ PEER<br>EDUCATOR 14	OUTREACH/ PEER<br>EDUCATOR 14
			CAMPAIGN 15	CAMPAIGN 15	CAMPAIGN 15
			OTHER PUBLIC<br>__________ 16<br>(SPECIFY)	OTHER PUBLIC<br>__________ 16<br>(SPECIFY)	OTHER PUBLIC<br>__________ 16<br>(SPECIFY)
			**PRIVATE MEDICAL SECTOR**	**PRIVATE MEDICAL SECTOR**	**PRIVATE MEDICAL SECTOR**
			PRIVATE HOSPITAL/<br>CLINIC 21	PRIVATE HOSPITAL<br>CLINIC 21	PRIVATE HOSPITAL/<br>CLINIC 21
			PHARMACY/CHEMICAL/<br>DRUG STORE . . . 22	PHARMACY/CHEMICAL/<br>DRUG STORE . . . 22	PHARMACY/CHEMICAL/<br>DRUG STORE 22
			OTHER PRIVATE<br>MEDICAL<br>__________ 26<br>(SPECIFY)	OTHER PRIVATE<br>MEDICAL<br>__________ 26<br>(SPECIFY)	OTHER PRIVATE<br>MEDICAL<br>__________ 26<br>(SPECIFY)
			**OTHER SOURCE**	**OTHER SOURCE**	**OTHER SOURCE**
			NGO/CBAs 31	NGO/CBAs 31	NGO/CBAs 31
			SHOP/MARKET . . . 32	SHOP/MARKET . . . 32	SHOP/MARKET 32
			STREET VENDOR . 33	STREET VENDOR . 33	STREET VENDOR . . 33
			PETROL STATION/<br>MOBILE MART . . . 34	PETROL STATION/<br>MOBILE MART . . . 34	PETROL STATION/<br>MOBILE MART 34
			PRIMARY SCHOO . 35	PRIMARY SCHOO . 35	PRIMARY SCHOOL . . 35
			OTHER 36<br>__________<br>(SPECIFY)	OTHER 36<br>__________<br>(SPECIFY)	OTHER 36<br>__________<br>(SPECIFY)
			DON'T KNOW . . . 98	DON'T KNOW . . . 98	DON'T KNOW 98

		NET #1	NET #2	NET #3
006	How much did it cost you to obtain this net?<br><br>RECORD '00.00' IF FREE OF CHARGE.	COST IN CEDIS<br><br>[ ][ ].[ ][ ]<br><br>DON'T KNOW . . . 9998	COST IN CEDIS<br><br>[ ][ ].[ ][ ]<br><br>DON'T KNOW . . . 9998	COST IN CEDIS<br><br>[ ][ ].[ ][ ]<br><br>DON'T KNOW . . . 9998
007	OBSERVE OR ASK THE BRAND/ TYPE OF MOSQUITO NET.<br><br>IF BRAND IS UNKNOWN AND YOU CANNOT OBSERVE THE NET, SHOW PICTURES OF TYPICAL NET TYPES/BRANDS TO RESPONDENT.	**LONG-LASTING INSECTICIDE-** TREATED NET (LLIN)<br>OLYSET . . . . 10<br>PERMANET . . . 11<br>INTERCEPTOR . 12<br>NETPROTECT . 13<br>DURANET . . . . . 14<br>LIFE NET . . . . . 15<br>DAWA PLUS . . . 16<br>MAGNET . . . . . 17<br>YORKOOL . . . . . 18<br>OTHER/ DK BRAND . . . 19<br>(SKIP TO 134)<br><br>**'PRETREATED' NET**	**LONG-LASTING INSECTICIDE-** TREATED NET (LLIN)<br>OLYSET . . . . 10<br>PERMANET . . . 11<br>INTERCEPTOR . 12<br>NETPROTECT . 13<br>DURANET . . . . . 14<br>LIFE NET . . . . . 15<br>DAWA PLUS . . . 16<br>MAGNET . . . . 17<br>YORKOOL . . . . . 18<br>OTHER/ DK BRAND . . 19<br>(SKIP TO 134)<br><br>**'PRETREATED' NET**	**LONG-LASTING INSECTICIDE-** TREATED NET (LLIN)<br>OLYSET . . . . 10<br>PERMANET . . . . . 11<br>INTERCEPTOR . . 12<br>NETPROTECT . . 13<br>DURANET . . . . . 14<br>LIFE NET . . . . . 15<br>DAWA PLUS . . . 16<br>MAGNET . . . . . 17<br>YORKOOL . . . . . 18<br>OTHER/ DK BRAND . . 19<br>(SKIP TO 134)<br><br>**'PRETREATED' NET**

		OTHER/<br>DK BRAND . . . 26<br>(SKIP TO 134)<br>**OTHER**<br>LOCALLY SEWN<br>NETS . . . . 31<br>OTHER BRAND . . 96<br>DK BRAND 98	OTHER/<br>DK BRAND . . . 26<br>(SKIP TO 134)<br>**OTHER**<br>LOCALLY SEWN<br>NETS . . . . 31<br>OTHER BRAND . . . 96<br>DK BRAND 98	OTHER/<br>DK BRAND . . . . . 26<br>(SKIP TO 134)<br>**OTHER**<br>LOCALLY SEWN<br>NETS . . . . 31<br>OTHER BRAND . . . . 96<br>DK BRAND 98
008	When you got the net, was it already treated with an insecticide to kill or repel mosquitoes?	YES . . . . 1<br>NO . . . . 2<br>NOT SURE 8	YES . . . . 1<br>NO . . . . 2<br>NOT SURE 8	YES . . . . 1<br>NO . . . . 2<br>NOT SURE 8
009	Did anyone sleep under this mosquito net last night?	YES . . . . 1<br>NO . . . . 2<br>(SKIP TO 136)<br>NOT SURE 8	YES . . . . 1<br>NO . . . . 2<br>(SKIP TO 136)<br>NOT SURE 8	YES . . . . 1<br>NO . . . . 2<br>(SKIP TO 136)<br>NOT SURE 8

		NET #1	NET #2	NET #3
010	Who slept under this mosquito net last night?<br><br>RECORD THE PERSON'S NAME AND LINE NUMBER FROM THE HOUSEHOLD SCHEDULE.	NAME_____________<br>LINE NO. . . . . . [ ][ ]<br>NAME_____________<br>LINE NO. . . . . . [ ][ ]	NAME_____________<br>LINE NO. . . . . . [ ][ ]<br>NAME_____________<br>LINE NO. . . . . . [ ][ ]	NAME_____________<br>LINE NO. . . . . . [ ][ ]<br>NAME_____________<br>LINE NO. . . . . . [ ][ ]

		NAME_____________<br><br>LINE NO. . . . . . [ ][ ]<br><br>NAME_____________<br><br>LINE NO. . . . . . [ ][ ]	NAME_____________<br><br>LINE NO. . . . . . [ ][ ]<br><br>NAME_____________<br><br>LINE NO. . . . . . [ ][ ]	NAME_____________<br><br>LINE NO. . . . . . [ ][ ]<br><br>NAME_____________<br><br>LINE NO. . . . . . [ ][ ]
011		GO BACK TO 010 FOR NEXT NET; OR, IF NO MORE NETS, GO TO 012A.	GO BACK TO 010 FOR NEXT NET; OR, IF NO MORE NETS, GO TO 012A.	GO TO 010 IN FIRST COLUMN OF A NEW QUESTIONNAIRE; OR, IF NO MORE NETS, GO TO 012A.

NO.	QUESTIONS AND FILTERS	CODING CATEGORIES	SKIP
012A	During the last 12 months has any member of your household disposed of any treated net?	YES . . . . 1<br>NO . . . . 2<br>DON'T KNOW 3	(SKIP TO 136E)
012B	How did you dispose of your last treated mosquito net?	BURNED . . . . 1<br>BURIED . . . . 2<br>GARBAGE OR REFUSE DUMP . . . . 3<br>REUSED FOR OTHER PURPOSE . . . . 4<br>OTHER ______________________ 6<br>(SPECIFY)<br>DON'T KNOW 8	

012C	How long did you use the net before disposing of it?	LESS THAN 2 YEARS	1
		2-4 YEARS	2
		MORE THAN 4 YEARS	3
		DON'T KNOW	8
012D	What was the main reason for disposing of this net?	TORN	1
		COULD NOT REPEL MOSQUITOS ANYMORE .	2
		GOT A NEW ONE	3
		OTHER (SPECIFY) ………………………………	6
		DON'T KNOW	8
012A	Have you ever heard about malaria?	YES	1
		NO	2
		DON'T KNOW	3

			YES	NO
013	In the past 6 months, have you seen or heard any messages about malaria:		YES	NO
	a) On the television?	TELEVISION	1	2
	b) On the radio?	RADIO	1	2
	c) In a newspaper or magazine?	NEWSPAPER/ MAGAZINE	1	2
	d) From a poster?	POSTER	1	2
	e) From leaflets or brochures?	LEAFLET/BROCHURE	1	2
	f) From a health worker?	HEALTH WORKER	1	2
	g) From a Community volunteer/CHW/CBA?	VOLUNTEER	1	2
	h) Anyone/anywhere else? Where/Whom?	OTHER	1	2
