# Supplementary material for: Insecticide-treated net (ITN) use, factors associated with non-use of ITNs, and occurrence of sand flies in three communities with reported cases of cutaneous leishmaniasis in Ghana
Source: PLoS One. 2021 Dec 16;16(12):e0261192. doi: 10.1371/journal.pone.0261192 (PMC8675665; doi:10.1371/journal.pone.0261192)
Supplement: Attachment — Submitted filename: New Response comments-28102021.docx [file pone.0261192.s004.docx]

Dear Dr. Akuffo,

We've checked your submission and before we can proceed, we need you to address the following issues:

1. We note that Figure 1 in your submission contain map images which may be copyrighted. All PLOS content is published under the Creative Commons Attribution License (CC BY 4.0), which means that the manuscript, images, and Supporting Information files will be freely available online, and any third party is permitted to access, download, copy, distribute, and use these materials in any way, even commercially, with proper attribution. For these reasons, we cannot publish previously copyrighted maps or satellite images created using proprietary data, such as Google software (Google Maps, Street View, and Earth). For more information, see our copyright guidelines: http://journals.plos.org/plosone/s/licenses-and-copyright.

We require you to either (1) present written permission from the copyright holder to publish these figures specifically under the CC BY 4.0 license, or (2) remove the figures from your submission:

A. You may seek permission from the original copyright holder of Figure 1 to publish the content specifically under the CC BY 4.0 license.

We recommend that you contact the original copyright holder with the Content Permission Form (http://journals.plos.org/plosone/s/file?id=7c09/content-permission-form.pdf) and the following text:
“I request permission for the open-access journal PLOS ONE to publish XXX under the Creative Commons Attribution License (CCAL) CC BY 4.0 (http://creativecommons.org/licenses/by/4.0/). Please be aware that this license allows unrestricted use and distribution, even commercially, by third parties. Please reply and provide explicit written permission to publish XXX under a CC BY license and complete the attached form.”

Please upload the completed Content Permission Form or other proof of granted permissions as an "Other" file with your submission.

In the figure caption of the copyrighted figure, please include the following text: “Reprinted from [ref] under a CC BY license, with permission from [name of publisher], original copyright [original copyright year].

Response: The Figure 1 was created by the study team using ArcGIS software version 10.4.1. As a result, Figure 1 can be made freely available online without any restriction or copyright issues being raised. For the sake of emphasis, Figure was not downloaded from any existing source. It is entirely a creation of the study team. The study team had licence to use the ArcGIS software and therefore does not require additional permission to publish Figure 1.

2. Please ensure that you refer to Figure xxxxx in your text as, if accepted, production will need this reference to link the reader to the figure.

Response: This is noted and has been corrected.
